# S2-alar-iliac screw and S1 pedicle screw fixation for the treatment of non-osteoporotic sacral fractures: a finite element study

**DOI:** 10.1186/s13018-021-02805-8

**Published:** 2021-10-30

**Authors:** Jianxiong Zheng, Xiaoreng Feng, Jie Xiang, Fei Liu, Frankie K. L. Leung, Bin Chen

**Affiliations:** 1 Division of Orthopaedics and Traumatology, Department of Orthopaedics, Nanfang Hospital, Southern Medical University, No. 1838 North Guangzhou Avenue, Guangzhou, 510515 China; 2 Department of Orthopaedics and Traumatology, Queen Mary Hospital, The University of Hong Kong, 5/f, Professorial Block, Pok Fu Lam Road, Pok Fu Lam, Hong Kong SAR China; 3 Department of Orthopaedics and Traumatology, Yangjiang People’s Hospital, Yangjiang, China

**Keywords:** S2-alar-iliac screw, Sacral fracture, Pelvic ring injury, Sacropelvic fixation, Biomechanics

## Abstract

**Background:**

Five different sacral fracture fixation methods were compared using finite element (FE) analysis to study their biomechanical characteristics.

**Methods:**

Denis type I sacral fractures were created by FE modeling. Five different fixation methods for the posterior pelvic ring were simulated: sacroiliac screw (SIS), lumbopelvic fixation (LPF), transiliac internal fixator (TIFI), S2-alar-iliac (S2AI) screw and S1 pedicle screw fixation (S2AI-S1) and S2AI screw and contralateral S1 pedicle screw fixation (S2AI-CS1). Four different loading methods were implemented in sequence to simulate the force in standing, flexion, right bending and left twisting, respectively. Vertical stiffness, relative displacement and change in relative displacement were recorded and analyzed.

**Results:**

As predicted by the FE model, the vertical stiffness of the five groups in descending order was S2AI-S1, SIS, S2AI-CS1, LPF and TIFI. In terms of relative displacement, groups S2AI-S1 and S2AI-CS1 displayed a lower mean relative displacement, although group S2AI-CS1 exhibited greater displacement in the upper sacrum than group S2AI-S1. Group SIS displayed a moderate mean relative displacement, although the displacement of the upper sacrum was smaller than the corresponding displacement in group S2AI-CS1, while groups LPF and TIFI displayed larger mean relative displacements. Finally, in terms of change in relative displacement, groups TIFI and LPF displayed the greatest fluctuations in their motion, while groups SIS, S2AI-S1 and S2AI-CS1 displayed smaller fluctuations.

**Conclusion:**

Compared with SIS, unilateral LPF and TIFI, group S2AI-S1 displayed the greatest biomechanical stability of the Denis type I sacral fracture FE models. When the S1 pedicle screw insertion point on the affected side is damaged, S2AI-CS1 can be used as an appropriate alternative to S2AI-S1.

**Supplementary Information:**

The online version contains supplementary material available at 10.1186/s13018-021-02805-8.

## Background

Sacroiliac screws (SIS), lumbopelvic fixation (LPF) and transiliac internal fixators (TIFI) have been widely used clinically as the three conventional methods of posterior pelvic ring injury treatment. Nevertheless, each method has limitations [1–5]. Sacroiliac screw placement with a residual displacement of 10 mm or more can endanger adjacent neural and vascular structures [1]. In addition, the reported incidences of sacroiliac screw loosening and screw failure are as high as 17.3% and 11.8%, respectively [2]. Several postoperative complications are present in the clinical practice of LPF, such as incision infection, screw prominence and implant failure [3, 4]. Long-term fixation of the lumbar vertebrae increases the likelihood of lower back discomfort [4]. TIFI has the advantages of being minimally invasive and displays a low postoperative infection rate, although it still cannot prevent local skin discomfort caused by screw protrusion [5].

To address the problems caused by the protrusion of the iliac screws, the S2-alar-iliac (S2AI) technique has been proposed to replace the iliac screw in lumbosacropelvic fixation [6, 7]. The S2AI screw is placed 15 mm deeper than the iliac screw, which makes it less prominent [6]. Biomechanical testing of S2AI screw fixation resulted in comparable stability versus traditional iliac screw fixation [8]. Furthermore, the S2AI screw technique has fewer postoperative complications, compared to the iliac screw technique [9]. However, the S2AI screw technique is mainly used in spine-related operations [10, 11], and few studies [12, 13] that applied the S2AI screw technique to treat posterior pelvic ring injuries exist. In these studies [12, 13], the lumbar spine was fixed, limiting normal lumbosacral movement.

For Denis type I sacral fractures, the lumbosacral joint is considered to be preserved and therefore has a certain degree of stability [14]. The purpose of surgery is only to resolve pelvic instability. We propose the S2AI screw and the ipsilateral S1 pedicle screw fixation (S2AI-S1) as a new internal fixation method to address problems in LPF, such as high invasiveness to soft tissue, the limitations to the mobility of lumbar vertebrae and adjacent segment disease [15]. This short-segment fixation method maintains the mobility of the lumbar spine and resolves concerns about adjacent segment disease. In addition, we propose another configuration that involves S2AI screw fixation with the contralateral S1 pedicle screw (S2AI-CS1). However, it is still unclear whether the new internal fixation methods can meet the requirements of biomechanical stability for the treatment of sacral fractures. Furthermore, the differences in biomechanical stability between new internal fixation methods and traditional fixation methods remain to be elucidated.

The aim of this study was to investigate the biomechanical stability of S2AI-S1, S2AI-CS1, SIS, LPF and TIFI for the treatment of non-osteoporotic sacral fractures (Denis type I) through finite element (FE) analysis.

## Materials and methods

CT data of the lumbar spine and pelvis were collected from a healthy male volunteer (30 years old, 175 cm, 70 kg, normal bone structure, no tumors, no deformities, no lumbar spine and pelvic structural damage). The CT imaging data were processed using Mimics 21.0 (Materialise, Belgium), Geomagic Studio 2013 (Geomagic, USA) and Solidworks 2017 (Dassault Systèmes, France) software to construct a model of the lumbar spine and entire pelvis. The interpelvic ligaments included iliolumbar ligament, anterior sacroiliac ligament, long posterior sacroiliac ligament (LPSL), short posterior sacroiliac ligament (SPSL), interosseous sacroiliac ligament, sacrospinous ligament, sacrotuberous ligament, superior pubic ligament and arcuate pubic ligament, all of which were simulated as spring structures in the finite element analysis software, ANSYS 17.0 (ANSYS, USA). The values for Young’s modulus (MPa) and Poisson's ratio (*u*) of cortical bone, cancellous bone, articular cartilage, intervertebral and interpubic disk tissue, and titanium metal were derived from the literature [16, 17]. The properties of ligaments are expressed in stiffness (N/mm) [18] (Table 1). The thicknesses of the cortical bone and endplates in the FE model were assumed to be 1.3 mm and 0.8 mm, respectively [19]. The thicknesses of sacral cartilage and iliac cartilage in the sacroiliac joint were 1.8 mm and 0.9 mm, respectively, and the gap between the two cartilages was 0.3 mm [20]. All bony parts and implants were meshed using a 10-node tetrahedron element.

**Table 1:** Material properties used in the finite element models

Materials	Young’s modulus (MPa)	Poisson’s ratio (*u*)	References
Titanium screw/rod/plate	110,000	0.3	[16]
Cortical bone (pelvis)	17,000	0.3	[17]
Cancellous bone (pelvis)	100	0.2	[17]
Cortical bone (lumbar)	12,000	0.3	[17]
Cancellous bone (lumbar)	100	0.2	[17]
Posterior bony elements	3500	0.25	[16]
Articular cartilage	10	0.4	[16]
Bony endplate	1000	0.4	[16]
Cartilage endplate	25	0.25	[16]
Nucleus pulposus	1	0.499	[16]
Matrix of annulus fibrosus	4.2	0.45	[16]
Fibers of annulus fibrosus	450	0.3	[16]
Interpubic disk	5	0.45	[16]

Ligament	Stiffness (N/mm)	Number of elements	References
Anterior sacroiliac ligament	700	10 × 2	[18]
Posterior sacroiliac ligament (long)	1000	4 × 2	[18]
Posterior sacroiliac ligament (short)	400	10 × 2	[18]
Interosseous sacroiliac ligament	2800	4 × 2	[18]
Sacrospinous ligament	1400	5 × 2	[18]
Sacrotuberous ligament	1500	5 × 2	[18]
Superior pubic ligament	500	1 × 1	[18]
Accurate pubic ligament	500	1 × 1	[18]
Iliolumbar ligament	1000	4 × 2	[18]

In the FE models, the contact condition between the sacroiliac joint cartilage was defined as friction contact, and the friction coefficient was 0.015 [21]. Meanwhile, a friction coefficient of 0.3 was applied between the interaction surfaces of fractures [21]. The interfaces between the superior and inferior articular processes, between the plates/bars and the screws, and between the screw thread part and bone were modeled with a bonded contact.

Denis type I models (right side) were obtained, respectively, by grid line segmentation. The ligaments were injured as follows: Denis type I sacral fracture accompanied injuries of the LPSL and part of the SPSL. The anterior pelvic ring destruction resulted in injuries of the interpubic disk, superior pubic ligament and arcuate pubic ligament.

**Fig. 1:**
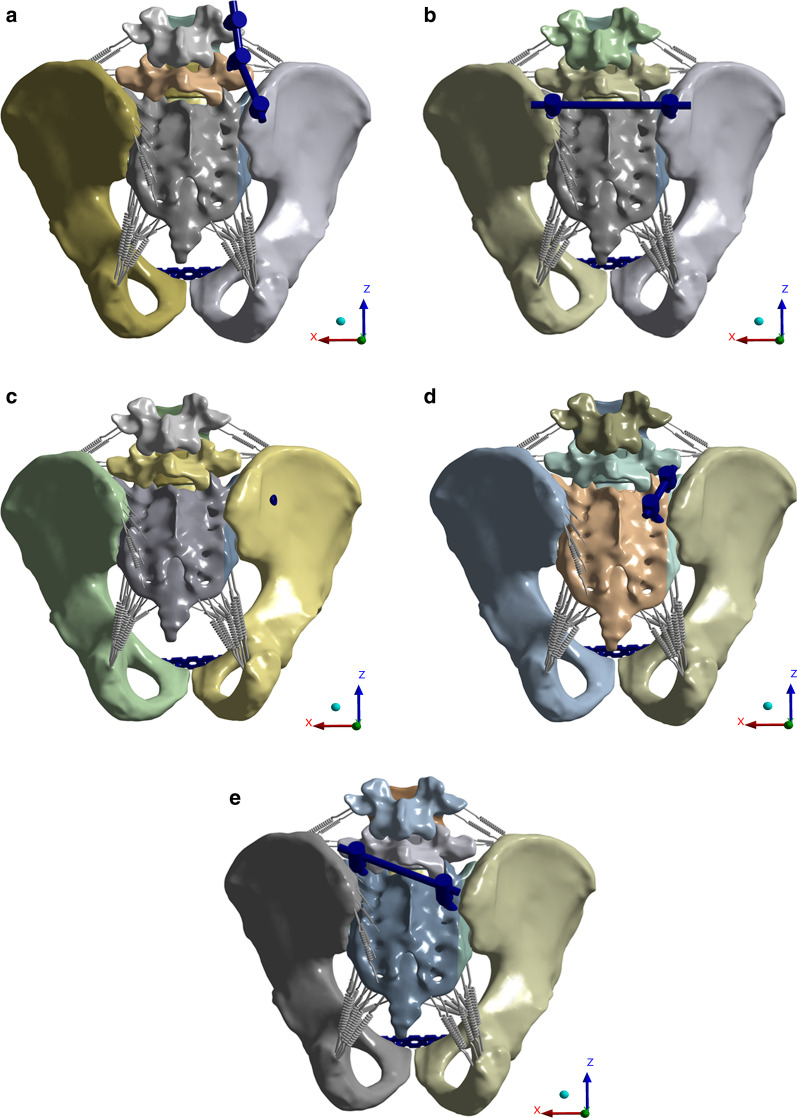
FE models of five internal fixation methods. **a** LPF model. **b** TIFI model. **c** SIS model. **d** S2AI-S1 model. **e** S2AI-CS1 model

The anterior pelvic ring was fixed with a plate, and the methods of fixing the posterior pelvic ring were as follows: In the SIS group, a half-thread hollow screw (7.3 mm × 75 mm) was placed horizontally into the S1 segment; in the S2AI-S1 and S2AI-CS1 groups, the S2AI screw (7.5 mm × 80 mm) was inserted according to the method in study [22] and then connected to the S1 pedicle screw with a rod; unilateral LPF was formed by connecting pedicle screws L4–L5 and an iliac screw (7.5 mm × 80 mm); and TIFI was formed by connecting iliac screws on both sides. In the FE model, the long screws close to the fracture line were defined as the main screws, while the others as secondary screws (Fig. 1a–e).

A standing posture with two legs was simulated as the boundary condition: the acetabulum on both sides was fixed in all directions. A vertical force of 500 N [21] was applied on the top surface of the L4 as the loading condition, to simulate the upper body weight. According to the three-column spinal theory, the upper endplate of L4 shares 85% of the load, and the bilateral superior articular facets of L4 share 15% of the load [16]. In addition, flexion was simulated by a 500 N vertical load and a 10 Nm moment in the forward sagittal direction [21]. Right bending was simulated by a 500 N vertical load and a 10 Nm moment in the right lateral direction. Finally, left twisting was simulated by a 500 N vertical load and a 10 Nm moment in the left horizontal rotation direction.

**Fig. 2:**
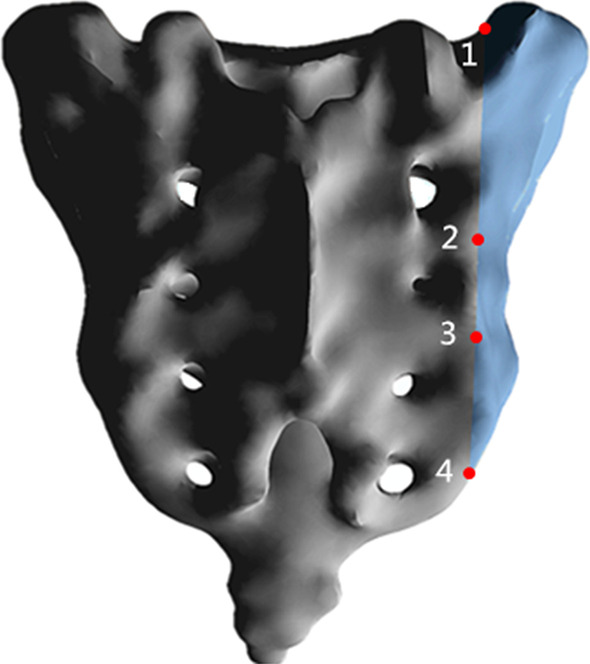
Four observation points in the FE model. Points 1 and 4 were located at the upper and lower ends of the sacrum, respectively. Point 2 was located at the intersection of the horizontal midline between S1 and S2 posterior sacral foramen and the fracture line. Point 3 was located at the intersection of the horizontal midline between S2 and S3 posterior sacral foramen and the fracture line

The vertical stiffness of each group was calculated based on the vertical displacement of the center point of the upper surface of S1 (vertical stiffness = 500 N/vertical displacement). Four pairs of points (Fig. 2) on both sides of the fracture line were selected to calculate the relative displacement (RD) of the fracture. The RD was used to evaluate the stability effect of each group of internal fixation on the sacral fracture model. The stability of the fixation is increased for smaller RD. In addition, changes in the relative displacement caused by three types of motion with respect to standing were calculated, to evaluate the range of fluctuation in the relative displacement of motion conditions relative to standing conditions (Additional file 1).

## Results

### Mesh convergence study

The number of elements used in the FE models of the SIS, LPF, TIFI, S2AI-S1 and S2AI-CS1 groups were 766,579, 772,157, 754,849, 751,032 and 757,516, respectively. A mesh convergence study was conducted and the appropriate mesh resolution for the FE model was determined from the influence of the maximum von Mises stress on bone. Doubling the number of elements in the FE models changed the maximum von Mises stress on the bone by 2.22%, 1.85%, 2.07%, 1.95% and 1.99% for the SIS, LPF, TIFI, S2AI-S1 and S2AI-CS1 groups, respectively, indicating that the original mesh size in the FE models satisfied the accuracy requirements of the analysis.

### Validation of the pelvic FE models

Under 500 N vertical loads, the FE predicted maximum compressive displacement (0.640 to 1.136 mm) of the intact pelvic model was consistent with the corresponding experiment-measured peak compressive displacements (0.973 to 1.550 mm) reported by Comstock et al. [23] under the same loading condition. In addition, similar to the simulative conditions in the study by Lu et al. [19], a 6.5 mm sacroiliac screw was used to fix the Tile C type pelvic ring injury. The average displacement of the observation site of the sacral wing edge was 1.5820 mm, which was close to the 2.0 mm value reported in the literature. Using experimental conditions for the hip bones positioned upside down similar to those reported by Dalstra et al. [24], the FE model predicted von Mises stresses (3.259 to 10.747 MPa under a 600 N load) for the hip bone material that were consistent with the corresponding experimentally measured von Mises stresses using eight strain gauges (0.712 to 7.641 MPa experiencing loads of 600 N).

### Vertical stiffness of the FE models

The vertical displacement (*Z*-axis) of the center point of the upper surface of the S1 vertebral body of the five groups in descending order were TIFI, LPF, S2AI-CS1, SIS and S2AI-S1 (Table 2). According to the stiffness calculation formula, the vertical stiffness of the five groups in descending order were S2AI-S1, SIS, S2AI-CS1, LPF and TIFI, as predicted by the FE model.

**Table 2:** Vertical displacement and maximum von Mises stress distribution of five groups

Groups	Vertical displacement (mm)*	The maximum von Mises stress in the implant (MPa)	The maximum von Mises stress in the bone around the screw (MPa)	
			Main screw**	Secondary screw
LPF	0.9861	97.354	26.810	10.402
TIFI	1.0694	65.580	21.390	0.638
SIS	0.7119	165.770	31.955	–
S2AI-S1	0.6864	55.262	26.695	26.304
S2AI-CS1	0.7491	63.589	36.839	7.715

*The vertical displacement (*Z*-axis) of the center point of the upper surface of the S1 vertebral body

**In the LPF and TIFI groups, the main screw is the right iliac screw, while in the S2AI-S1 and S2AI-CS1 groups, the main screw is the S2AI screw. The screws in each group except one main screw are secondary screws

### Maximum von Mises stress in the implant and bone around the screw

**Fig. 3:**
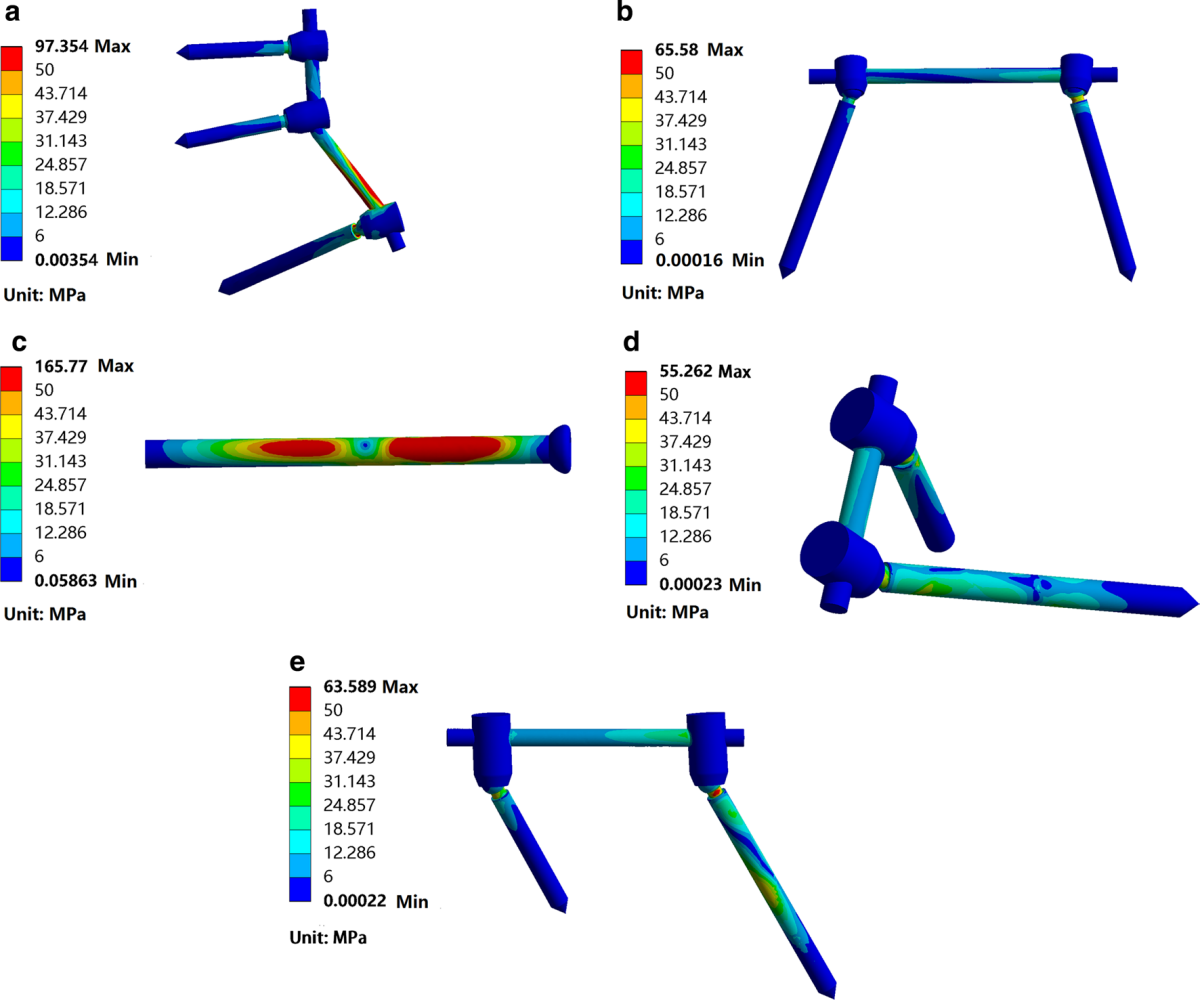
Stress distribution of the five implants under standing condition. **a** LPF group, the maximum equivalent stress was located at the connecting rod near the iliac screw head. **b** TIFI group, the maximum equivalent stress was located at the junction of the right iliac screw head and shaft. **c** SIS group, the maximum equivalent stress was located at the middle of the screw. **d** S2AI-S1 group, the maximum equivalent stress was located at the junction of the S1 pedicle screw head and shaft. **e** S2AI-CS1 group, the maximum equivalent stress was located at junction of the S2AI screw head and shaft

Using 500 N vertical loading, the maximum von Mises stresses of the implants of the five groups in descending order were SIS, LPF, TIFI, S2AI-CS1 and S2AI-S1 (Fig. 3a–e). The maximum von Mises stress values of all implants were less than the yield stress of titanium [25]. This indicates that implant failure due to exceeding the maximum von Mises stress of implants in the five groups was not predicted. Under 500 N vertical stress, the maximum von Mises stresses of the bone around the main screw of the five groups in descending order were S2AI-CS1, SIS, LPF, S2AI-S1 and TIFI. In addition, the maximum von Mises stress values of the bone around the secondary screw of the four groups in descending order were S2AI-S1, LPF, S2AI-CS1 and TIFI. The maximum von Mises stress of the bone around the screws in all models was located within the cortical bone but lower than the yield strength of cortical bone [26]. This indicates that secondary fracture due to the maximum von Mises stress of the sacrum in the five groups was not predicted (Table 2).

### Relative displacement and change in relative displacement

**Fig. 4:**
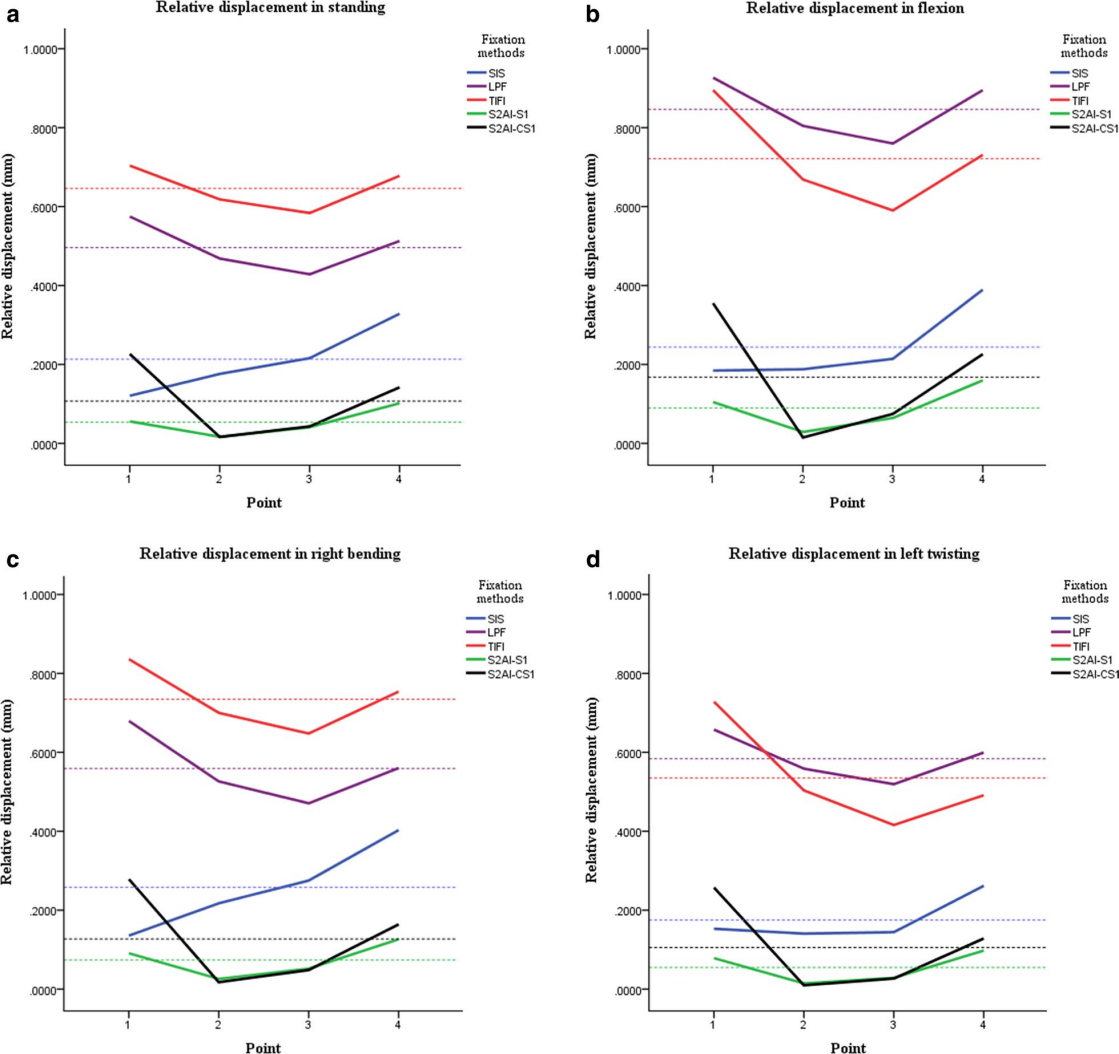
Relative displacement distribution diagrams in four different conditions. The dotted line of each color corresponds to the solid line of each color. The dotted line represents the average relative displacement. **a** Relative displacement in standing. **b** Relative displacement in flexion. **c** Relative displacement in right bending. **d** Relative displacement in left twisting

Using four different loading methods, the mean RD (mm) of the five groups in descending order were TIFI, LPF, SIS, S2AI-CS1 and S2AI-S1 (except that the mean RD of LPF in flexion and left twisting was greater than in TIFI) (Fig. 4a–d). The RD of the S2AI-CS1 group at point 1 was greater than that of the S2AI-S1 and SIS groups, while the RD at points 2–4 was similar to that of the S2AI-S1 group, but smaller than in the SIS group (Additional file 2, Additional file 3, Additional file 4, Additional file 5).

Compared with standing, the change in relative displacement of the SIS, S2AI-S1 and S2AI-CS1 groups for three different movements fluctuated less than in LPF and TIFI. For the LPF group, the change in relative displacement during flexion was larger, while in right bending and left twisting the changes were smaller. For the TIFI group, the change in relative displacement was larger in flexion and left twisting, while it was smaller in right bending (Table 3).

**Table 3:** Change in relative displacement of the five fixation methods

Motion	Fixation method	The change in relative displacement (mm)			
		1	2	3	4
Flexion—standing	LPF	0.3670	0.3455	0.3373	0.3909
	TIFI	0.2906	0.1348	0.0855	0.0630
	SIS	0.0811	0.0291	0.0290	0.0722
	S2AI-S1	0.0492	0.0125	0.0245	0.0581
	S2AI-CS1	0.1308	0.0101	0.0423	0.0878
Right bending—standing	LPF	0.1181	0.0693	0.0526	0.0761
	TIFI	0.1583	0.1147	0.0981	0.1074
	SIS	0.0165	0.0452	0.0624	0.0758
	S2AI-S1	0.0352	0.0101	0.0134	0.0257
	S2AI-CS1	0.0534	0.0015	0.0059	0.0223
Left twisting—standing	LPF	0.1015	0.0915	0.0911	0.1041
	TIFI	0.3292	0.2684	0.2612	0.2001
	SIS	0.0608	0.0659	0.0965	0.0758
	S2AI-S1	0.0255	0.0073	0.0201	0.0123
	S2AI-CS1	0.0418	0.0092	0.0208	0.0139

*LPF* lumbopelvic fixation, *TIFI* transiliac internal fixator, *SIS* sacroiliac screw, *S2AI-S1* S2-alar-iliac screw and S1 pedicle screw fixation, *S2AI-CS1* S2-alar-iliac screw and contralateral S1 pedicle screw fixation, *1–4*, respectively, represent 4 observation points

## Discussion

The incidence of non-osteoporotic sacral fractures has been reported as 2.1 cases per 100,000 of the population and usually occurs when young individuals have a traffic accident or fall from a height [27]. Due to the complex local anatomy and unique biomechanics, the fixation of sacral fractures remains a challenge [28]. A key aspect of sacral fracture repair is sufficient stability to counterbalance translational and rotational forces in the vertical and horizontal directions [3]. In addition, to improve the patient quality of life following surgery, sacral implants should also minimize adverse effects on patients. In the present study, static FE analysis methodology that is widely accepted by the community was used to evaluate the stability of five internal fixation methods on sacral fracture. The simulation results showed that the new internal fixation methods for the treatment of Denis type I sacral fractures can meet safety and stability requirements.

In the biomechanical experiment of artificial bone models (Denis type II sacral fractures), the posterior pelvic ring was fixed with SIS and unilateral LPF, respectively. At 4000 cycles (500 N), the relative displacements of the SIS group and the unilateral LPF group were 4.02 ± 1.18 mm and 15.59 ± 4.00 mm (*P* < 0.005), respectively. Although the Denis type I fracture model was used in this study, stability in the SIS group was higher than in the unilateral LPF group. This points to the consistency between the simulation results and biomechanical experiments. In an FE analysis study, Song et al. found that unilateral LPF could not provide sufficient horizontal and rotational stability for patients with unilateral sacral fractures [29]. In the present study, the change in relative displacement of the unilateral LPF fluctuated greatly in flexion compared with standing, indicating that unilateral LPF is potentially unstable. In another study, Vigdorchik et al. found that the stiffness of the SIS was greater than the pedicle screw construct, namely TIFI, under the sacroiliac joint injury model [30]. Although a Denis type I fracture model was used, the stiffness of SIS, which was greater than TIFI in our study, was consistent with the result of Vigdorchik et al.

A higher maximum von Mises stress value indicates that the model has a higher risk of implant failure [31]. According to the results of the simulation (except in the SIS gorup), the LPF group displayed a greater risk of implant failure, occurring at the connection between the iliac screw and rod. This confirms previous clinical studies, which found that implant failure usually manifests as disengagement of the screw from the rod connector [32]. The TIFI and S2AI-CS1 groups displayed a moderate risk of implant failure at the shaft and junction of the head and main screw. The S2AI-S1 group exhibited a lower risk of implant failure, which also occurred at the shaft and the junction of the head and secondary screw.

Higher stresses on the bone around the screw may lead to screw loosening and secondary fractures [21]. Based on the results of the simulation, the SIS group has a higher risk of screw loosening than the LPF group. Furthermore, the main screws were predicted to have a greater risk of loosening than the secondary screws. For the main screws, the S2AI-CS1 and SIS groups had the highest risk of screw loosening, the S2AI-S1 and LPF groups displayed a moderate risk of screw loosening, while the TIFI group had the lowest risk of screw loosening. For the secondary screws, the S1 pedicle screw in the S2AI-S1 group had a higher risk of loosening than other screws. Therefore, it is believed that the disadvantage of S2AI-S1 is a relatively increased risk of loosening of the secondary screw. Comparing the S2AI-S1 group with the S2AI-CS1 group, the ipsilateral S1 pedicle screw had a greater influence in reducing the maximum von Mises stress for the S2AI screw than the contralateral S1 pedicle screw.

According to the mean RD values, the overall stability of the five internal fixation methods in the FE models in descending order was: S2AI-S1, S2AI-CS1, SIS, LPF and TIFI. Considering that unilateral LPF and TIFI are insufficient for stability of the posterior pelvic ring, the addition of a sacroiliac screw to unilateral LFP or TIFI to enhance stability is considered necessary, as demonstrated in previous studies [3, 33]. The RD of different points reveals that a fracture is stabler the closer it is to a screw (S2AI screw/Sacroiliac screw). This indicates that a single-segment S1 sacroiliac screw provides limited stability for the lower sacrum. A number of researchers have suggested adding an S2 sacroiliac screw for biplanar stability [19, 34]. Since the S2AI-CS1 group had greater RD at point 1 and point 4 than the S2AI-S1 group, the former was slightly less stable, when used in sacral fractures, than the latter. In addition, although the overall stability of the S2AI-CS1 group for sacral fractures was superior to that of SIS, the stability of the upper sacrum was slightly worse.

The change in relative displacement reveals the principal reason for the difference in stability. TIFI and unilateral LPF are methods that can achieve indirect stabilization of sacral fractures through a screw–rod system, while SIS, S2AI-S1 and S2AI-CS1 can provide direct fixation of sacral fractures using screws. According to the AO principles of fracture management, fixation of the fracture interface using screws could create a preload [35]. This preload would compress the fracture and prevent separation, while friction between the fracture surfaces and between the screw and bone would oppose displacement, due to shear [35]. By comparing the relative changes in displacement for three types of motion, groups TIFI and LPF displayed greater fluctuations (relative stability) during motion, while the SIS, S2AI-S1 and S2AI-CS1 groups exhibited smaller fluctuations (absolute stability). This indicates that the SIS, S2AI-S1 and S2AI-CS1 groups can provide a stabler healing environment for patients with Denis type I sacral fractures, than the TIFI and LPF groups.

This study confirmed the role of the new internal fixation in the stability of Denis I sacral fractures, providing a biomechanical basis for its clinical application. The invasiveness of LPF and problems with the fixation range (i.e., limitation of mobility of lumbar vertebrae, adjacent segment disease and the need for implant removal) [15] suggest that S2AI-S1 may be a beneficial method for internal fixation. Compared with TIFI, S2AI-S1 is not only expected to improve biomechanical stability, but also eliminate the problem of screw protrusion. Although S2AI-S1 may be more invasive than SIS [36], the former has better biomechanical stability and a lower risk of screw loosening than the latter. Considering that S2AI-CS1 is slightly less stable in the upper sacrum and that the main screw has a higher risk of loosening, such fixation is not suggested as the first choice. When the S1 pedicle screw insertion point on the affected side is damaged, S2AI-CS1 can be used as a good alternative to S2AI-S1.

## Limitations

Nevertheless, potential limitations related to this study should be noted. First, these simulation results are only based on a FE analysis of static linear displacement, while screw loosening was not considered. The stability of S2AI-S1 needs to be further verified by cyclic loading experiments on cadaver bone models. Second, the screw in this experiment used simplified processing to reduce the calculation time and analysis error due to stress concentration. However, to obtain accurate stress distribution on the screw and the bone around the screw, the screw thread should be considered in the FE model [37]. Third, in this study, only the main ligaments were simulated, but in practice, other ligaments and muscles may still play a role [38]. Therefore, differences between our simulation data and cadaver bone model data may exist. Finally, the pelvic data of a healthy 30-year-old male used to represent the patient population are also a limitation, and no anatomical differences between individuals were considered. In addition, this study only simulated non-osteoporotic sacral fractures, and the impact of osteoporosis on internal fixation was not considered. However, for a more comprehensive understanding of the safety and stability of new internal fixation methods, a model of poor bone quality should also be established.

## Conclusion

Compared with SIS, unilateral LPF and TIFI, S2AI-S1 displayed the best biomechanical stability of the Denis type I sacral fracture FE models. When the S1 pedicle screw insertion point on the affected side is damaged, S2AI-CS1 can be used as a appropriate alternative to S2AI-S1.

## Supplementary Information

**Additional file 1: **The calculation formula of relative displacement and change in relative displacement.

**Additional file 2: **The relative displacement in standing.

**Additional file 3: **The relative displacement in flexion.

**Additional file 4: **The relative displacement in right bending.

**Additional file 5: **The relative displacement in left twisting.

## Data Availability

All data generated or analyzed during this study are included in this published article and its additional information files.
