# Supplementary material for: S2-alar-iliac screw and S1 pedicle screw fixation for the treatment of non-osteoporotic sacral fractures: a finite element study
Source: J Orthop Surg Res. 2021 Oct 30;16:651. doi: 10.1186/s13018-021-02805-8 (PMC8557573; doi:10.1186/s13018-021-02805-8)
Supplement: Supplementary file 2 — Additional file 2: The relative displacement in standing. [file 13018_2021_2805_MOESM2_ESM.pdf]

Additional file 2
Relative displacement in standing

**LPF**	1	2	3	4
Xa<br>Xb	-0.1106<br>-0.1212	-0.0640<br>-0.0784	-0.0538<br>-0.0606	0.0288<br>-0.0369
RDx(leftward)	0.0106	0.0144	0.0068	0.0657
Ya<br>Yb	1.2156<br>1.3080	0.9235<br>0.8271	0.8167<br>0.6584	0.5109<br>0.1935
RDy(backward)	-0.0924	0.0964	0.1583	0.3174
Za<br>Zb	-1.2215<br>-0.6543	-1.4035<br>-0.9455	-1.5082<br>-1.1101	-1.4974<br>-1.0999
RDz(upward)	-0.5672	-0.4580	-0.3981	-0.3975
**RD**	**0.5748**	**0.4683**	**0.4285**	**0.5129**
**TIFI**	1	2	3	4
Xa<br>Xb	-0.1284<br>-0.1702	-0.0700<br>-0.1231	-0.0540<br>-0.1081	0.0389<br>-0.0635
RDx(leftward)	0.0418	0.0531	0.0541	0.1024
Ya<br>Yb	1.2626<br>1.2771	0.9735<br>0.7923	0.8670<br>0.6224	0.5675<br>0.1541
RDy(backward)	-0.0145	0.1812	0.2446	0.4134
Za<br>Zb	-1.3119<br>-0.6095	-1.4918<br>-0.9031	-1.5962<br>-1.069	-1.5862<br>-1.0587
RDz(upward)	-0.7024	-0.5887	-0.5272	-0.5275
**RD**	**0.7038**	**0.6182**	**0.5837**	**0.6780**
**SIS**	1	2	3	4
Xa<br>Xb	-0.0275<br>-0.0305	-0.0470<br>-0.1728	-0.0510<br>-0.2294	-0.0496<br>-0.3530
RDx(leftward)	0.0030	0.1258	0.1784	0.3034
Ya<br>Yb	1.304<br>1.3048	0.9191<br>0.8817	0.7768<br>0.7348	0.3803<br>0.3296
RDy(backward)	-0.0008	0.0374	0.042	0.0507
Za<br>Zb	-0.8692<br>-0.7486	-1.1196<br>-1.0023	-1.2599<br>-1.1456	-1.2513<br>-1.1360
RDz(upward)	-0.1206	-0.1173	-0.1143	-0.1153
**RD**	**0.1206**	**0.1760**	**0.2160**	**0.3285**
**S2AI-S1**	1	2	3	4
Xa<br>Xb	0.0498<br>0.0493	-0.0377<br>-0.0436	-0.0656<br>-0.0797	-0.1069<br>-0.1077
RDx(leftward)	0.0005	0.0059	0.0141	0.0008
Ya	1.2341	0.8525	0.7139	0.3372

Yb	1.2687	0.8369	0.6767	0.2354
RDy(backward)	-0.0346	0.0156	0.0372	0.1018
Za<br>Zb	-0.7829<br>-0.7391	-1.0104<br>-1.0091	-1.1526<br>-1.1627	-1.1493<br>-1.1507
RDz(upward)	-0.0438	-0.0013	0.0101	0.0014
**RD**	**0.0558**	**0.0167**	**0.0410**	**0.1018**
**S2AI-CS1**	1	2	3	4
Xa<br>Xb	0.0127<br>-0.0323	-0.0233<br>-0.0273	-0.0344<br>-0.0528	-0.0554<br>-0.0558
RDx(leftward)	0.0450	0.0040	0.0184	0.0004
Ya<br>Yb	1.2735<br>1.4442	0.9052<br>0.8935	0.7490<br>0.7100	0.3453<br>0.2038
RDy(backward)	-0.1707	0.0117	0.0390	0.1415
Za<br>Zb	-0.8992<br>-0.7574	-1.1009<br>-1.0904	-1.2643<br>-1.2645	-1.2584<br>-1.2469
RDz(upward)	-0.1418	-0.0105	0.0002	-0.0115
**RD**	**0.2264**	**0.0162**	**0.0431**	**0.1420**

Point a is located inside the fracture line, and point b is located outside the fracture line. Xa and Xb respectively represent the displacement of the two points relative to the origin on the X axis. Ya and Yb respectively represent the displacement of the two points on the Y axis relative to the origin. Za and Zb respectively represent the displacement of the two points on the Z axis relative to the origin.

LPF: Lumbopelvic fixation ;

TIFI: Transiliac internal fixator ;

SIS: sacroiliac screw ;

S2AI-S1: S2-alar-iliac screw and S1 pedicle screw fixation ;

S2AI-CS1: S2-alar-iliac screw and contralateral S1 pedicle screw fixation.

RDx: The relative displacement of the two points a, b on the X axis. Leftward is a positive value

RDy: The relative displacement of the two points a, b on the Y axis. Backward is a positive value

RDz: The relative displacement of the two points a, b on the Z axis .Upward is a positive value

RD: The total relative displacement of two points a, b in the three-dimensional direction
