# Supplementary material for: S2-alar-iliac screw and S1 pedicle screw fixation for the treatment of non-osteoporotic sacral fractures: a finite element study
Source: J Orthop Surg Res. 2021 Oct 30;16:651. doi: 10.1186/s13018-021-02805-8 (PMC8557573; doi:10.1186/s13018-021-02805-8)
Supplement: Supplementary file 3 — Additional file 3: The relative displacement in flexion. [file 13018_2021_2805_MOESM3_ESM.pdf]

Additional file 3
Relative displacement in flexion

**LPF**	1	2	3	4
Xa<br>Xb	-0.1152<br>-0.1465	-0.0280<br>-0.0397	-0.0027<br>-0.0052	0.1107<br>0.0941
RDx(leftward)	0.0313	0.0117	0.0025	0.0166
Ya<br>Yb	-0.1098<br>-0.0926	0.1818<br>-0.0832	0.2765<br>-0.0795	0.5241<br>-0.0702
RDy(backward)	-0.0172	0.2650	0.3560	0.5943
Za<br>Zb	-0.9148<br>0.0110	-0.7434<br>0.0161	-0.6518<br>0.0195	-0.6498<br>0.0192
RDz(upward)	-0.9258	-0.7595	-0.6713	-0.669
**RD**	**0.9265**	**0.8045**	**0.7599**	**0.8950**
**TIFI**	1	2	3	4
Xa<br>Xb	-0.1084<br>-0.1780	-0.0506<br>-0.0937	-0.0391<br>-0.0624	0.0791<br>0.0027
RDx(leftward)	0.0696	0.0431	0.0233	0.0764
Ya<br>Yb	-0.2582<br>0.0032	0.0586<br>-0.0110	0.1579<br>-0.0157	0.4251<br>-0.0289
RDy(backward)	-0.2614	0.0696	0.1736	0.4540
Za<br>Zb	-0.8813<br>-0.0281	-0.7005<br>-0.0369	-0.6052<br>-0.0417	-0.6093<br>-0.0413
RDz(upward)	-0.8532	-0.6636	-0.5635	-0.568
**RD**	**0.8951**	**0.6686**	**0.5901**	**0.7311**
**SIS**	1	2	3	4
Xa<br>Xb	0.0284<br>0.0318	-0.0176<br>-0.1315	-0.0249<br>-0.1851	-0.0387<br>-0.3898
RDx(leftward)	-0.0034	0.1139	0.1602	0.3511
Ya<br>Yb	-0.0781<br>-0.0164	0.1141<br>0.0735	0.1649<br>0.1061	0.2968<br>0.1954
RDy(backward)	-0.0617	0.0406	0.0588	0.1014
Za<br>Zb	-0.4232<br>-0.2494	-0.3329<br>-0.1892	-0.2868<br>-0.1573	-0.2931<br>-0.1587
RDz(upward)	-0.1738	-0.1437	-0.1295	-0.1344
**RD**	**0.1845**	**0.1878**	**0.2142**	**0.3894**
**S2AI-S1**	1	2	3	4
Xa<br>Xb	0.1012<br>0.0949	-0.0057<br>-0.0132	-0.0392<br>-0.0525	-0.0967<br>-0.1039
RDx(leftward)	0.0063	0.0075	0.0133	0.0072
Ya	-0.1546	0.0372	0.0911	0.2460

Yb	-0.0884	0.0096	0.0300	0.0865
RDy(backward)	-0.0662	0.0276	0.0611	0.1595
Za<br>Zb	-0.2778<br>-0.1968	-0.1538<br>-0.1495	-0.1124<br>-0.1276	-0.1241<br>-0.1276
RDz(upward)	-0.081	-0.0043	0.0152	0.0035
**RD**	**0.1048**	**0.0289**	**0.0643**	**0.1597**
**S2AI-CS1**	1	2	3	4
Xa<br>Xb	0.0770<br>-0.0054	0.0267<br>0.0259	0.0102<br>-0.0004	-0.0399<br>-0.0396
RDx(leftward)	0.0824	0.0008	0.0106	-0.0003
Ya<br>Yb	-0.1863<br>0.0948	0.0610<br>0.0461	0.1094<br>0.0423	0.2579<br>0.0323
RDy(backward)	-0.2811	0.0149	0.0671	0.2256
Za<br>Zb	-0.3760<br>-0.1749	-0.2053<br>-0.2038	-0.1725<br>-0.2033	-0.1826<br>-0.1963
RDz(upward)	-0.2011	-0.0015	0.0308	0.0137
**RD**	**0.3553**	**0.0150**	**0.0746**	**0.2260**

Point a is located inside the fracture line, and point b is located outside the fracture line. Xa and Xb respectively represent the displacement of the two points relative to the origin on the X axis. Ya and Yb respectively represent the displacement of the two points on the Y axis relative to the origin. Za and Zb respectively represent the displacement of the two points on the Z axis relative to the origin.

LPF: Lumbopelvic fixation ;

TIFI: Transiliac internal fixator ;

SIS: sacroiliac screw ;

S2AI-S1: S2-alar-iliac screw and S1 pedicle screw fixation ;

S2AI-CS1: S2-alar-iliac screw and contralateral S1 pedicle screw fixation.

RDx: The relative displacement of the two points a, b on the X axis. Leftward is a positive value

RDy: The relative displacement of the two points a, b on the Y axis. Backward is a positive value

RDz: The relative displacement of the two points a, b on the Z axis .Upward is a positive value

RD: The total relative displacement of two points a, b in the three-dimensional direction
