# Supplementary material for: S2-alar-iliac screw and S1 pedicle screw fixation for the treatment of non-osteoporotic sacral fractures: a finite element study
Source: J Orthop Surg Res. 2021 Oct 30;16:651. doi: 10.1186/s13018-021-02805-8 (PMC8557573; doi:10.1186/s13018-021-02805-8)
Supplement: Supplementary file 4 — Additional file 4: The relative displacement in right bending. [file 13018_2021_2805_MOESM4_ESM.pdf]

Additional file 4
Relative displacement in right bending

**LPF**	1	2	3	4
Xa<br>Xb	-0.1412<br>-0.1829	-0.0608<br>-0.0736	-0.0398<br>-0.0409	0.0751<br>0.0635
RDx(leftward)	0.0417	0.0128	0.0011	0.0116
Ya<br>Yb	1.0621<br>1.2218	0.8335<br>0.7645	0.7483<br>0.6045	0.4998<br>0.1627
RDy(backward)	-0.1597	0.0690	0.1438	0.3371
Za<br>Zb	-1.2698<br>-0.6107	-1.4097<br>-0.8881	-1.4931<br>-1.0448	-1.4826<br>-1.0353
RDz(upward)	-0.6591	-0.5216	-0.4483	-0.4473
**RD**	**0.6795**	**0.5263**	**0.4708**	**0.5602**
**TIFI**	1	2	3	4
Xa<br>Xb	-0.1720<br>-0.2385	-0.0638<br>-0.1596	-0.0328<br>-0.1325	0.1134<br>-0.0628
RDx(leftward)	0.0665	0.0958	0.0997	0.1762
Ya<br>Yb	1.0186<br>1.1284	0.8252<br>0.6964	0.7518<br>0.5449	0.5410<br>0.1278
RDy(backward)	-0.1098	0.1288	0.2069	0.4132
Za<br>Zb	-1.3591<br>-0.5328	-1.4759<br>-0.7945	-1.5478<br>-0.9423	-1.5386<br>-0.9331
RDz(upward)	-0.8263	-0.6814	-0.6055	-0.6055
**RD**	**0.8362**	**0.7001**	**0.6476**	**0.7539**
**SIS**	1	2	3	4
Xa<br>Xb	-0.0579<br>-0.0682	-0.0551<br>-0.2238	-0.0479<br>-0.2870	-0.0371<br>-0.4150
RDx(leftward)	0.0103	0.1687	0.2391	0.3779
Ya<br>Yb	1.1762<br>1.1745	0.8546<br>0.8104	0.7337<br>0.6848	0.3929<br>0.3383
RDy(backward)	0.0017	0.0442	0.0489	0.0546
Za<br>Zb	-0.8339<br>-0.6987	-1.0463<br>-0.9165	-1.1658<br>-1.0388	-1.1591<br>-1.0302
RDz(upward)	-0.1352	-0.1298	-0.1270	-0.1289
**RD**	**0.1356**	**0.2174**	**0.2751**	**0.4030**
**S2AI-S1**	1	2	3	4
Xa<br>Xb	0.0214<br>0.0157	-0.0412<br>-0.0495	-0.0577<br>-0.0775	-0.0809<br>-0.0817
RDx(leftward)	0.0057	0.0083	0.0198	0.0008
Ya	1.1166	0.7937	0.6713	0.3437

Yb	1.1726	0.7704	0.6235	0.2176
RDy(backward)	-0.0560	0.0233	0.0478	0.1261
Za<br>Zb	-0.7790<br>-0.7078	-0.9645<br>-0.9572	-1.0925<br>-1.0967	-1.0905<br>-1.0835
RDz(upward)	-0.0712	-0.0073	0.0042	-0.0070
**RD**	**0.0908**	**0.0258**	**0.0519**	**0.1263**
**S2AI-CS1**	1	2	3	4
Xa<br>Xb	-0.0057<br>-0.0737	-0.0233<br>-0.0270	-0.0252<br>-0.0436	-0.0335<br>-0.0333
RDx(leftward)	0.0680	0.0037	0.0184	-0.0002
Ya<br>Yb	1.0965<br>1.3045	0.8101<br>0.7972	0.6769<br>0.6322	0.3404<br>0.1766
RDy(backward)	-0.2080	0.0129	0.0447	0.1638
Za<br>Zb	-0.8697<br>-0.6973	-1.015<br>-1.0037	-1.1573<br>-1.1591	-1.1533<br>-1.1413
RDz(upward)	-0.1724	-0.0113	0.0018	-0.012
**RD**	**0.2786**	**0.0175**	**0.0484**	**0.1642**

Point a is located inside the fracture line, and point b is located outside the fracture line. Xa and Xb respectively represent the displacement of the two points relative to the origin on the X axis. Ya and Yb respectively represent the displacement of the two points on the Y axis relative to the origin. Za and Zb respectively represent the displacement of the two points on the Z axis relative to the origin.

LPF: Lumbopelvic fixation ;

TIFI: Transiliac internal fixator ;

SIS: sacroiliac screw ;

S2AI-S1: S2-alar-iliac screw and S1 pedicle screw fixation ;

S2AI-CS1: S2-alar-iliac screw and contralateral S1 pedicle screw fixation.

RDx: The relative displacement of the two points a, b on the X axis. Leftward is a positive value

RDy: The relative displacement of the two points a, b on the Y axis. Backward is a positive value

RDz: The relative displacement of the two points a, b on the Z axis .Upward is a positive value

RD: The total relative displacement of two points a, b in the three-dimensional direction
