# Supplementary material for: S2-alar-iliac screw and S1 pedicle screw fixation for the treatment of non-osteoporotic sacral fractures: a finite element study
Source: J Orthop Surg Res. 2021 Oct 30;16:651. doi: 10.1186/s13018-021-02805-8 (PMC8557573; doi:10.1186/s13018-021-02805-8)
Supplement: Supplementary file 5 — Additional file 5: The relative displacement in left twisting. [file 13018_2021_2805_MOESM5_ESM.pdf]

Additional file 5
Relative displacement in left twisting

LPF	1	2	3	4
Xa<br>Xb	-0.0515<br>-0.1096	-0.0474<br>-0.0672	-0.0599<br>-0.0607	0.0123<br>-0.0048
RDx(leftward)	0.0581	0.0198	0.0008	0.0171
Ya<br>Yb	1.0689<br>1.1318	0.8258<br>0.6957	0.7351<br>0.5429	0.4812<br>0.1219
RDy(backward)	-0.0629	0.1301	0.1922	0.3593
Za<br>Zb	-1.2314<br>-0.5795	-1.3871<br>-0.8442	-1.4762<br>-0.9938	-1.4638<br>-0.9843
RDz(upward)	-0.6519	-0.5429	-0.4824	-0.4795
**RD**	**0.6575**	**0.5586**	**0.5193**	**0.5994**
**TIFI**	1	2	3	4
Xa<br>Xb	-0.0272<br>-0.1252	-0.0640<br>-0.1043	-0.0973<br>-0.1007	-0.0202<br>-0.0731
RDx(leftward)	0.0980	0.0403	0.0034	0.0529
Ya<br>Yb	0.8055<br>1.1384	0.6232<br>0.6938	0.5519<br>0.5379	0.3625<br>0.1084
RDy(backward)	-0.3329	-0.0706	0.014	0.2541
Za<br>Zb	-1.2059<br>-0.5656	-1.3318<br>-0.8350	-1.4027<br>-0.9871	-1.3945<br>-0.9775
RDz(upward)	-0.6403	-0.4968	-0.4156	-0.417
**RD**	**0.7283**	**0.5034**	**0.4158**	**0.4912**
**SIS**	1	2	3	4
Xa<br>Xb	0.0417<br>0.0441	-0.0351<br>-0.1011	-0.0696<br>-0.1537	-0.0750<br>-0.3028
RDx(leftward)	-0.0024	0.066	0.0841	0.2278
Ya<br>Yb	1.0265<br>1.0841	0.7305<br>0.7201	0.6156<br>0.5941	0.3033<br>0.2468
RDy(backward)	-0.0576	0.0104	0.0215	0.0565
Za<br>Zb	-0.8483<br>-0.7067	-1.0466<br>-0.9229	-1.1608<br>-1.0455	-1.1524<br>-1.0365
RDz(upward)	-0.1416	-0.1237	-0.1153	-0.1159
**RD**	**0.1529**	**0.1406**	**0.1443**	**0.2618**
**S2AI-S1**	1	2	3	4

Xa<br>Xb	0.1313<br>0.1173	-0.0164<br>-0.0181	-0.0751<br>-0.0754	-0.1386<br>-0.1385
RDx(leftward)	0.014	0.0017	0.0003	-0.0001
Ya<br>Yb	1.0348<br>1.0857	0.7274<br>0.7146	0.6050<br>0.5765	0.2925<br>0.1952
RDy(backward)	-0.0509	0.0128	0.0285	0.0973
Za<br>Zb	-0.7496<br>-0.6915	-0.9306<br>-0.9240	-1.0587<br>-1.0571	-1.0562<br>-1.0462
RDz(upward)	-0.0581	-0.0066	-0.0016	-0.0100
**RD**	**0.0785**	**0.0145**	**0.0285**	**0.0978**
**S2AI-CS1**	1	2	3	4
Xa<br>Xb	0.0982<br>0.0213	0.0034<br>0.0035	-0.0376<br>-0.0393	-0.0820<br>-0.0813
RDx(leftward)	0.0769	-0.0001	0.0017	-0.0007
Ya<br>Yb	1.0308<br>1.2282	0.7527<br>0.7491	0.6179<br>0.5912	0.2840<br>0.1563
RDy(backward)	-0.1974	0.0036	0.0267	0.1277
Za<br>Zb	-0.8337<br>-0.6877	-0.9872<br>-0.9782	-1.1297<br>-1.1285	-1.1253<br>-1.1131
RDz(upward)	-0.1460	-0.0090	-0.0012	-0.0122
**RD**	**0.2573**	**0.0097**	**0.0268**	**0.1283**

Point a is located inside the fracture line, and point b is located outside the fracture line. Xa and Xb respectively represent the displacement of the two points relative to the origin on the X axis. Ya and Yb respectively represent the displacement of the two points on the Y axis relative to the origin. Za and Zb respectively represent the displacement of the two points on the Z axis relative to the origin.

LPF: Lumbopelvic fixation ;

TIFI: Transiliac internal fixator ;

SIS: sacroiliac screw ;

S2AI-S1: S2-alar-iliac screw and S1 pedicle screw fixation ;

S2AI-CS1: S2-alar-iliac screw and contralateral S1 pedicle screw fixation.

RDx: The relative displacement of the two points a, b on the X axis. Leftward is a positive value

RDy: The relative displacement of the two points a, b on the Y axis. Backward is a positive value

RDz: The relative displacement of the two points a, b on the Z axis .Upward is a positive value

RD: The total relative displacement of two points a, b in the three-dimensional direction
